# A Clinical Phrasebook in English and German

**Published:** 1853-05

**Authors:** 


*A Clinical Phrasebook, in English and German, containing the usual questions and answers employed in examining and prescribing for patients; questions in asking for and buying medicines, etc., etc., etc.* By Montgomery Johns, M. D. Philadelphia: Lindsay & Blakiston. 1853. 16mo., pp. 308.

This little work is designed to aid physicians and surgeons in hospitals, almshouses, and private practice; also druggists and pharmaceutists in dispensing their prescriptions. The necessity for it grows out of the large proportion of Germans in the population of many parts of our country. It will prove very convenient and useful to those who are so situated as to have occasion to hold intercourse with Germans, and who are imperfectly acquainted with the German language.

